# Computation predicts rapidly adapting mechanotransduction currents cannot account for tactile encoding in Merkel cell-neurite complexes

**DOI:** 10.1371/journal.pcbi.1006264

**Published:** 2018-06-29

**Authors:** Gregory J. Gerling, Lingtian Wan, Benjamin U. Hoffman, Yuxiang Wang, Ellen A. Lumpkin

**Affiliations:** 1 Department of Systems and Information Engineering, University of Virginia, Charlottesville, Virginia, United States of America; 2 Department of Mechanical and Aerospace Engineering, University of Virginia, Charlottesville, Virginia, United States of America; 3 Department of Biomedical Engineering, University of Virginia, Charlottesville, Virginia, United States of America; 4 Medical Scientist Training Program, Columbia University College of Physicians & Surgeons, New York, New York, United States of America; 5 Department of Physiology & Cellular Biophysics, Columbia University College of Physicians & Surgeons, New York, New York, United States of America; 6 Department of Dermatology, Columbia University College of Physicians & Surgeons, New York, New York, United States of America; Technische Universitat Chemnitz, GERMANY

## Abstract

Distinct firing properties among touch receptors are influenced by multiple, interworking anatomical structures. Our understanding of the functions and crosstalk of Merkel cells and their associated neurites—the end organs of slowly adapting type I (SAI) afferents—remains incomplete. Piezo2 mechanically activated channels are required both in Merkel cells and in sensory neurons for canonical SAI responses in rodents; however, a central unanswered question is how rapidly inactivating currents give rise to sustained action potential volleys in SAI afferents. The computational model herein synthesizes mechanotransduction currents originating from Merkel cells and neurites, in context of skin mechanics and neural dynamics. Its goal is to mimic distinct spike firing patterns from wildtype animals, as well as *Atoh1* knockout animals that completely lack Merkel cells. The developed generator function includes a Merkel cell mechanism that represents its mechanotransduction currents and downstream voltage-activated conductances (slower decay of current) and a neurite mechanism that represents its mechanotransduction currents (faster decay of current). To mimic sustained firing in wildtype animals, a longer time constant was needed than the 200 ms observed for mechanically activated membrane depolarizations in rodent Merkel cells. One mechanism that suffices is to introduce an ultra-slowly inactivating current, with a time constant on the order of 1.7 s. This mechanism may drive the slow adaptation of the sustained response, for which the skin’s viscoelastic relaxation cannot account. Positioned within the sensory neuron, this source of current reconciles the physiology and anatomical characteristics of *Atoh1* knockout animals.

## Author summary

Slowly-adapting type I (SAI) cutaneous afferents help us discriminate fine spatial details. Their physiology and anatomy are distinguished by their slow adaptation in firing to held stimuli and innervation of Merkel cells, respectively. How mechanotransduction currents in Merkel cells and sensory neurons combine to give rise to neural spike firing is unknown. In considering wildtype animals, as well as *Atoh1* conditional knockout animals that lack Merkel cells, this effort employs a computational modeling approach constrained by biological measurements. For the developed generator function to recapitulate firing responses across genotype, a previously unsuspected current source is required. Thus, the model makes specific predictions for future experimental studies.

## Introduction

A diverse array of touch receptors signal information from the periphery to the central nervous system, enabling the detection of objects we encounter at our skin surface [1,2]. In mammals, at least four classes of afferents serve to signal mechanical interactions, each tuned to extract specific features of a tactile stimulus. These classes of mechanosensory afferents encode tactile stimuli as trains of action potentials, or spikes, each with distinctive firing properties. One class of mechanosensitive neurons, myelinated Aβ slowly-adapting type I (SAI) afferents, are gentle touch receptors that encode edges and curvature. These mechanoreceptors localize to skin regions specialized for high tactile acuity, including fingertips, whisker follicles and touch domes. Several physiological characteristics distinguish SAI afferents from other mechanosensitive classes of neurons: 1) association with epidermal Merkel cells, 2) high frequency responses to moving stimuli, 3) slowly adapting responses to held stimuli, 4) irregular firing patterns with large variability in inter-spike intervals, and 5) sensitivities to a wide range of stimulus forces.

A common feature of mechanosensory neurons is specialized anatomical structures, termed end organs, which shape their neuronal outputs. Each myelinated SAI afferent branches and extends unmyelinated projections (neurites) that form synaptic-like contacts with Merkel cells (Merkel cell-neurite complexes). The SAI afferent’s end organ is a cluster of multiple Merkel cell-neurite complexes, which are required to produce canonical SAI firing patterns [3,4]. In response to mechanical stimulation, individual Merkel cell-neurite complexes produce a generator current in unmyelinated neurites, which are summed at a spike initiation sites located at myelinated branch points, termed heminodes, in the SAI afferent. At spike initiation sites, generator currents from clusters of Merkel cell-neurite complexes are converted to action potentials, which propagate towards the spinal cord.

Despite recent computational modeling to determine how the architecture of Merkel cell-neurite complexes governs SAI firing properties [5], it remains unknown how Merkel cells and neurites individually contribute to mechanically evoked responses in SAI afferents. It is experimentally difficult to directly measure the generator currents that Merkel cells and neurites individually create in response to mechanical stimulation, however recent physiological data suggests at least a two-component model. *Ex vivo* extracellular recordings of SAI afferents from a skin-specific *Atoh1* conditional knockout (*Atoh1*^*CKO*^) [3,4], which lack Merkel cells, exhibit truncated firing patterns during sustained mechanical stimulation. Additionally, similar recordings from mice whose Merkel cells lack *Piezo2* (*Piezo2*^*CKO*^) [3], a mechanically activated ion channel that is required for the intrinsic mechanosensitivity of Merkel cells, also showed truncated sustained firing. Finally, knockdown of Piezo2 in rat whisker follicles attenuate whisker-stimulated firing [6]. From these data emerge a two-receptor-site model of mechanotransduction at the Merkel cell-neurite complex: 1) Merkel cells are required for sustained firing in SAI afferents, while 2) neurites generate rapidly adapting firing to mechanical stimulation [3,7,8].

Here, we sought out to evaluate this conceptual framework by computationally modeling how Merkel cells and neurites individually contribute to SAI action potential volleys. Although previous groups have attempted to model SAI responses, the fundamental mechanics of these models are directly tied and fitted to a presented stimulus [9–15]. This critical confound obscures the individual biophysical interactions of Merkel cells and neurites, and reduces the physiological relevance of these models. The generator function developed herein is based on physiological data and is composed of a Merkel-cell mechanism (slower decay of current) and a neurite mechanism (faster decay of current). Numerical experiments, both at the level of the interaction of a single Merkel cell and neurite, and at the level of an entire end organ, demonstrate the impact of parameter changes, contributions, and interactions. Such computational experiments may help elucidate mechanisms of sensory encoding at the Merkel cell-neurite complex that govern tactile encoding and function, and in particular, reveal biological mechanisms, *in silico*, that are technically difficult to observe *in vivo*. Thus, the models generate specific predictions for future experimental studies.

## Results

To better understand how Merkel cells and neurites individually contribute to the generation of mechanically evoked SAI responses, we built a computational model to synthesize individual generator currents, in the context of skin mechanics and neural dynamics. Critical to this effort is a novel generator function, based on physiological responses rather than being directly tied and fitted to a stimulus. The generator function is composed of a Merkel-cell mechanism (slower decay of current) and a neurite mechanism (faster decay of current). The physiological data forming its basis were obtained from *in vitro* electrophysiological recordings of Merkel cells under current clamp and Piezo2-dependent currents in DRG neurons recorded under voltage clamp, which exhibit time constants on the order of 200 and 8 ms, respectively.

Our *in silico* analysis reveals that Piezo2-initiated slowly inactivating (SI) currents in Merkel cells and rapidly inactivating (RI) currents in neurites inadequately predict touch-evoked responses in Merkel-cell afferents. In one regard, this two-receptor-site model cannot reproduce the slow adaptation in spike firing of the sustained response, so characteristic of SAI afferents. To mimic sustained firing, a longer time constant is needed than the 200 ms observed for mechanically activated membrane depolarizations in rodent Merkel cells. One mechanism that suffices is to introduce an ultra-slowly inactivating (USI) current—a previously unsuspected component—with a time constant on the order of 1.7 s. This mechanism may drive the slow adaptation of the sustained response, for which the skin’s viscoelastic relaxation cannot account. In a second and perhaps more important regard, the positioning of the USI current source within the sensory neuron is essential in reconciling the physiology and anatomical characteristics of *Atoh1*^*CKO*^ animals, which lack Merkel cells. Together, a model incorporating SI, RI and USI mechanically activated currents, is capable of predicting firing patterns for wildtype Merkel-cell afferents, and replicating distinct, truncated firing patterns observed experimentally in recordings from epidermal-specific *Atoh1*^*CKO*^ mice.

### Computational model of a wildtype Merkel cell-neurite complex

**Fig 1:**
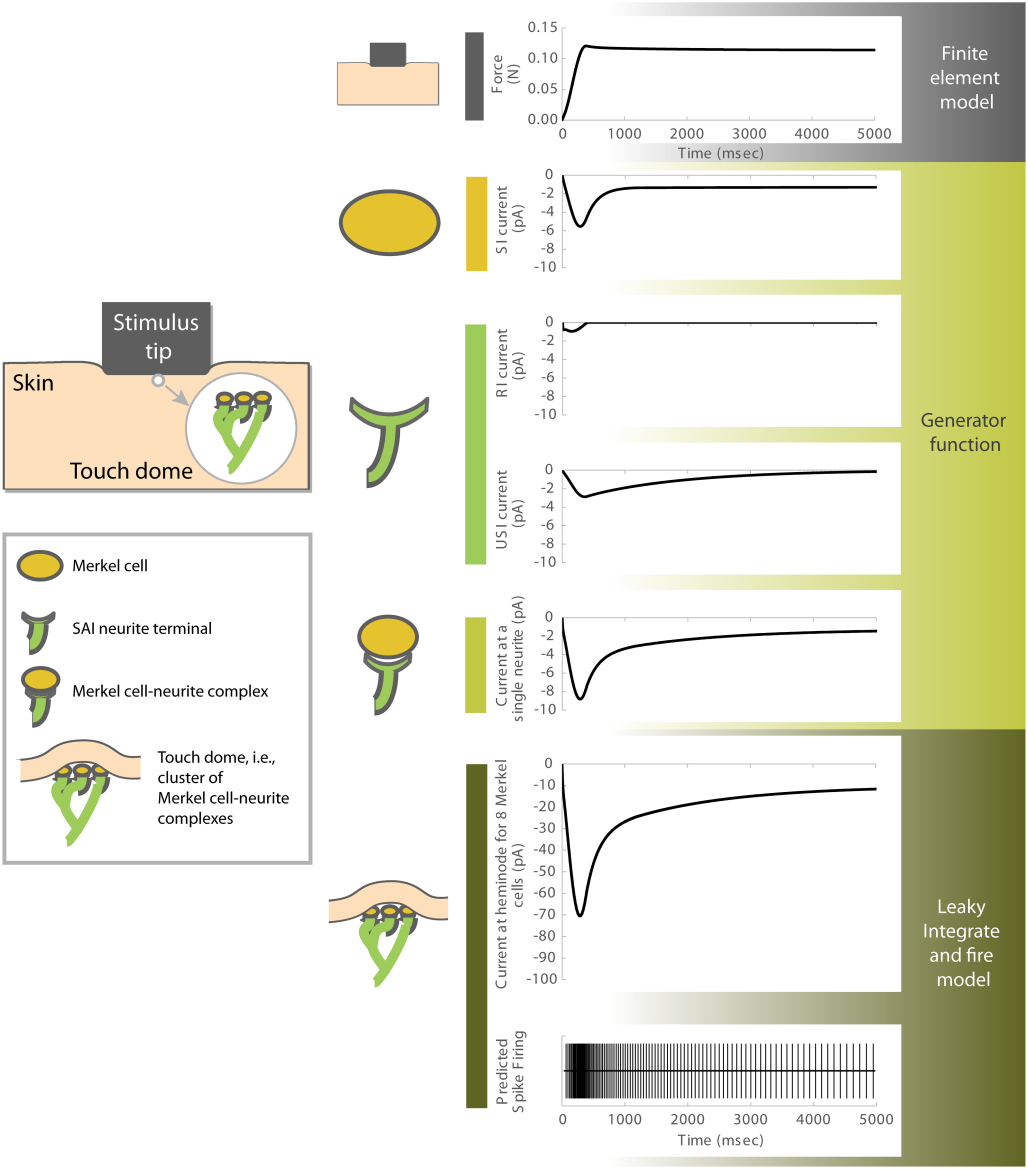
A computational model of mechanotransduction in the slowly adapting type I cutaneous afferent. Shown is current over a ramp and hold stimulus for multiple sub-components, including a slowly inactivating (SI) current modeled as originating from the Merkel cell and rapidly inactivating (RI) and ultra-slowly inactivating (USI) currents modeled as originating from the neurite terminal. All three currents are included within the generator function, which receives input of compressive stress from a finite element model. The finite element model takes probe force, as shown in the upper panel, as its input. The generated trace of compressive stress interior to the skin’s layers, as shown in Fig 2, exhibits time-dependent viscoelastic relaxation. The currents that the generator function represents, in modeling one Merkel cell—neurite complex, are summed across a cluster of eight Merkel cell-neurite complexes feeding a heminode. It is this current, upon entering into a leaky integrate and fire model, which gives rise to predicted spike firing times. Note that for the sake of the simulation here, the irregular inter-spike intervals were unimportant so noise was removed from the model.

Our computational model of mechanotransduction at the Merkel cell-neurite complex has three primary structural components: 1) a finite element model of skin mechanics, 2) a generator function for Merkel-cell and neurite based currents, and 3) a leaky integrate-and-fire (LIF) model to fire spikes at heminodes, which propagate to the afferent (Fig 1). Though the generator function is the focus of this work, its physiology is inseparably intertwined with the skin and arbor.

To comprehensively model mechanical forces in the skin, we utilized a finite element model of the skin’s layers that converts a displacement stimulus, with a linearly decelerating ramp-up (ramp phase), and a static hold (hold phase), into compressive stress within the skin’s layers (Fig 1, top). Each layer of the multi-layered model was represented by axisymmetric hybrid elements, comprising about 14,000 in number, of quasi-linear viscoelastic material using a two-term Prony series with Ogden and Neo-Hookean hyperelastic properties. This finite element model was used in a prior effort [16] and was directly informed by hyperelastic and viscoelastic properties based directly upon measurements of mouse skin [17,18]. In particular, the model includes mouse skin (all intact layers including epidermis, dermis and hypodermis, and excluding muscle) as well as a thin nylon mesh and elastomeric backing material. The skin specimen modeled was 380 μm thick, with the following mechanical properties (μ = 1.3 kPa, α = 7.9, τ_1_ = 0.08 s, τ_2_ = 1.2 s, G_1_ = 0.59, G_2_ = 0.10, G_∞_ = 0.31; note no unit indicates dimensionless quantity). The model has been rigorously validated [16]. In particular, force over time at the stimulus tip, in response to displacement clamped stimuli, have proven to be quite similar in magnitude and form to measurements made during physiological experiments.

In response to indentation by ramp-and-hold stimuli, compressive stress over time from the finite element model is passed to the generator function, which calculates generator current for one Merkel cell-neurite complex (Fig 1, middle). The generator function is composed of three individual currents, an SI current arising from Merkel-cell stimulation, and an RI and USI arising in neurites. We propose that the SI current is generated in the neurite downstream of activation of mechanosensitive ion-channels, such as Piezo2, in Merkel cells. The kinetics of this current was constrained by in vitro electrophysiological recordings in Merkel cells, as both Piezo-dependent mechanically activated currents and downstream voltage-activated calcium and potassium currents are expected to contribute to prolonged signaling between Merkel cells and sensory neurites. The SI current is sustained during the stimulus hold phase, with a gradual increase and large peak amplitude during the ramp phase of mechanical stimulation, and slow decay during the hold phase. The RI current is generated directly through mechanical activation of Piezo2 in neurites. Compared with the SI current, the RI current is more sensitive to changes in stimulation over the ramp phase, but has a low peak amplitude, and quickly decays to zero during the hold phase. The origin of the USI current is not tied to a specific molecular mechanism. This current has lowest sensitivity to the ramp phase, is of moderate peak amplitude, and has the slowest rate of decay during the hold phase.

The sum of all three currents is highly sensitive and has a large peak amplitude during the ramp phase. During the hold phase, this summed current decays with an initial slow phase (from peak value of stimulation to about 0.5 s afterwards) and a secondary ultra-slow phase (in response to the stress relaxation of the skin), maintaining a steady level through the late-hold phase (from 2 to 5 s of the stimulation). It is this generator current that underlies the model’s prediction of spike trains in SAI afferents. In particular, the summed current from each Merkel cell-neurite complex is multiplied by the number of complexes in a cluster, which predicts the total current entering an SAI afferent’s heminode. We then employed a LIF model to predict the required accumulated current to elicit action potential trains in SAI afferents (Fig 1, bottom). For the purposes of this simulation, the irregular inter-spike intervals were unimportant so noise was removed from the model, though present in a prior work [5]. Therefore, the output spike times are quite regular relative to actual SAI firing.

### Conceptual understanding of the generator function

The key conceptual contribution of the generator function is the linear convolution of internal skin stress over time with each of three physiologically-based sub-functions (SI, RI, and USI). This computational strategy enables the recent history of skin stress to be captured at any instantaneous time point. Each sub-function consists of a unique time constant and ratio of peak to steady state current. These parameters are directly derived from *in vitro* recordings of Merkel cells and SAI neurons in current clamp and voltage clamp mode, respectively (S1 Fig). Therefore, the modeled responses to mechanical step stimulation exhibit an instantaneous increase or decrease proportional to stress magnitude followed by exponential decay, as do the recorded responses. Electrophysiological recordings suggest that current in a neuron rapidly decays with a step stimulation, and we assume that a neurite behaves similarly. Therefore, when the three sub-functions sum together, the recent time history of stimulus magnitude and rate, as generator current, is carried to the present.

**Fig 2:**
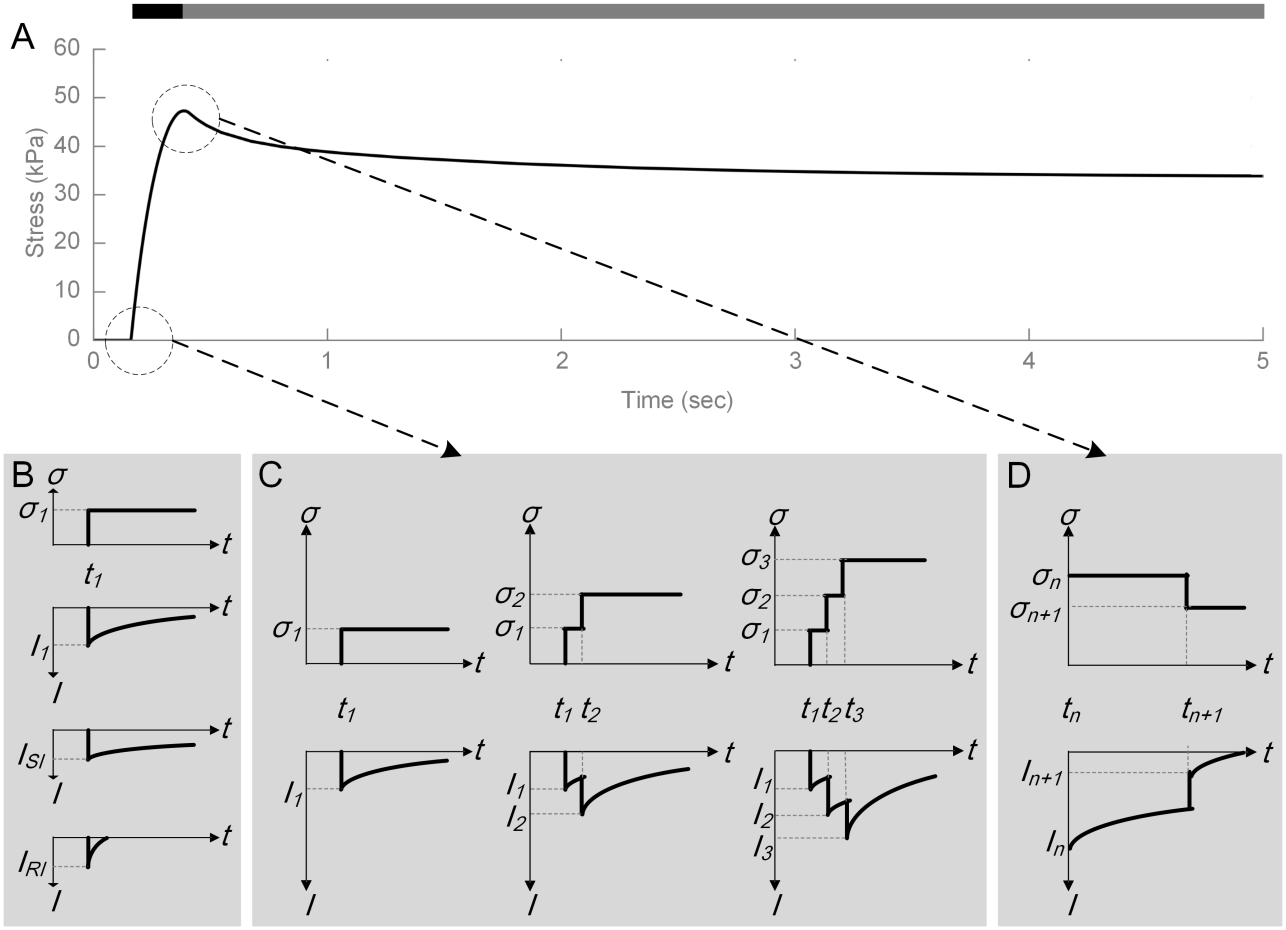
Conceptual view of the generator function. The diagrams demonstrate how small time constants of about 8 and 200 ms respectively for the SI and RI components are convolved together with stress in the skin to produce a composite current from which spike firing responses are ultimately derived. In (A) stress over time, under a displacement-controlled ramp-and-hold stimulation (top bar dark grey is ramp, light grey is hold), serves as the input to the generator function. Note the viscoelastic relaxation of skin stress over the stimulus hold. Then, in three cases with inputs of step stresses, (B) a single step increase in stress σ1 evokes current output I1, which is the sum of slowly inactivating current ISI and rapidly inactivating current IRI, (C) three sequentially delivered step stresses show that current decays but builds upon the prior magnitude, and (D) a single step decrease in stress from σn to σn+1 evokes an immediate decrease in current followed by a slower decay. Note that the ultra-slowly inactivating current is omitted to simplify the explanation of the concept. Also omitted for simplicity are the finite element and leaky integrate-and-fire model contributions.

The mechanics of the generator function are demonstrated by magnifying the view of compressive stress interior to the skin (Fig 2A), generated by a finite element model of skin mechanics in response to a ramp-and-hold stimulus, to show the impact of small, discretized step stresses (*σ*_*1*_, *σ*_*2*_ *– σ*_*n*_, etc.) in creating receptor current. In reality, stress output by the skin mechanics model is continuous but a discrete representation demonstrates the following concepts more readily. In Fig 2B, top, the generator function representing a single Merkel cell-neurite complex is input with one instantaneous step stress with value *σ*_*1*_ at time *t*_*1*_, where *σ*_*1*_ is a very small. In response, in Fig 2B, middle-top, the generator function produces an instantaneous current response (*I*_*1*_) linear to the stress value *σ*_*1*_ at time *t*_*1*_. Its value decreases over time to a stimulus held at that level of stress. *I*_*1*_ is composed of a fast-decaying current *I*_*RI*_ (Fig 2B, bottom) from the neurite mechanism, and a slow-decaying current *I*_*SI*_ (Fig 2B, middle-bottom) from the Merkel cell mechanism. Note that the USI current is omitted from Fig 2 to simplify the explanation of the concept. In Fig 2C, increasing stress over time and the generated current response is demonstrated. A second step stress at time *t*_*2*_ is added, making the total stress *σ*_*2*_ at time *t*_*2*_. In response, the current response increases to *I*_*2*_ from the *I*_*1*_ value which formed at *σ*_*1*_ and then began to decay over time. A third step stress at time *t*_*3*_ is then added, making the total stress *σ*_*3*_ at time *t*_*2*_. In response, the current response increases to *I*_*3*_ and will decay back to the baseline if there is no further stress input. Skin relaxation, which occurs at the end of the ramp phase of mechanical indentation, results in a small decrease in skin stress. In Fig 2D, we mimic the case of a decay in stress beginning at peak force. Assuming the stress decays from *σ*_*n*_ to *σ*_*n+1*_ at time *t*_*n+1*_, the current response drops immediately to *I*_*n+1*_, which is of a magnitude linearly related to the absolute change in stress, before continuing to decay in the fashion described in Fig 2B. Note that this overview of the generator function is detailed mathematically in *Methods*.

### Physiological data underlying the generator function

The generator function relies upon three free and five biologically derived parameters. Three of the biologically derived parameters, *τ*_*SI*_, *K*_*SI_Peak*_, and *K*_*SI_Steady*_, were directly fitted to data obtained from *in vitro* electrophysiological recordings of Merkel cells (S1A Fig) where the latter two represent the peak to steady-state ratio of decaying current. In particular, we assumed that Merkel cells and neurites communicate via synaptic transmission, where changes in membrane potential in Merkel cells are linearly related to post-synaptic current changes in neurites. Thus, current clamped recordings of Merkel cells most accurately reflect Merkel-cell dependent generator currents in neurites. In contrast, *τ*_*RI*_ was derived from data obtained from *in vitro* electrophysiological recordings of Piezo2-dependent currents in DRG neurons recorded under voltage clamp (S1B Fig; [19]). The *τ*_*USI*_ parameter was set to the time constant of 1.7 ms, found for putative LTMRs [20] [21] (see Discussion), and slightly model fit around that value. In contrast to the five biologically derived parameters, the three free parameters were fitted in the context of simulating an entire end organ *in silico*. These parameters set the relative magnitudes of the SI, RI, and USI currents. Their values were fitted in the context of the end-organ model so that firing rates predicted over the ramp-up, early-hold, and late-hold phases of the stimulus accurately recapitulated electrophysiological recordings from wildtype animals. Note that this overview of the physiological data is detailed further in *Methods*.

### Issue with fitting slow adaptation in firing response

**Fig 3:**
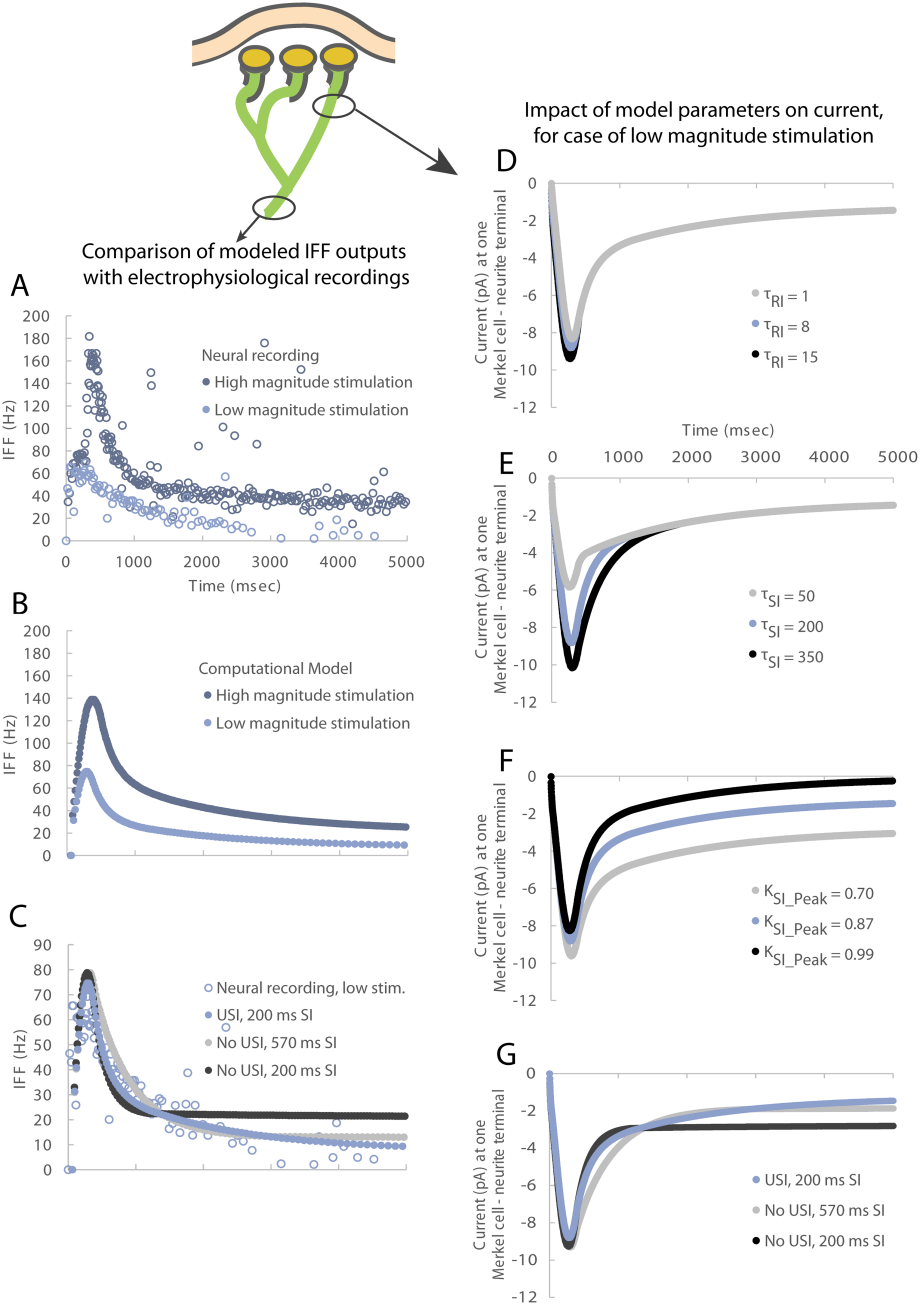
An ultra-slowly inactivating (USI) current is essential to drive the slow adaptation in firing in the sustained response. In contrast, neither the modification of the SI and RI currents of generator function, nor the skin’s viscoelastic relaxation, adequately account for the slow adaptation in firing in the sustained response. Instantaneous firing frequency (IFF) plots over time are generated (panel B) similar to those observed in electrophysiological recordings (panel A). The data for wildtype animals were originally reported in Maksimovic, et. al. 2014 [3]. The two traces per plot represent two magnitudes of ramp and hold stimulation. The need for the USI component is shown in panel C. Without the USI component, the output IFF reaches a plateau and does not adapt as is typically observed for SAI afferents. Another potential way to achieve adaptation is to increase the time constant on the SI component of the model from 200 to 570 ms. However, the duration of this time constant is well outside observed bounds. For the case of the low magnitude stimulus, in panels D—F, increasing generator function parameters τRI, τSI, and KSI_Peak can increase receptor current in ramp-up, early hold, and late hold phases, respectively, as well as their corresponding IFFs (S2 Fig). They do not however impact the plateau in the sustained hold. In particular, current traces with different (D) τRI values show the impact upon the peak current produced, (E) τSI values show the impact during the early hold phase, and (F) KSI_Peak values show the impact of modulating the steady state magnitude relative to the peak. Note that in panels D—F, the USI component is included. In panel G, currents are shown that correspond to IFFs in panel C. Data similar to panel C, but for the case of high magnitude stimulation, are given in S3 Fig. The tau values are in units of ms.

The model as described involves three generator currents. A previously published two-receptor-site model proposed that Merkel cells contribute SI currents and neurites contribute RI current to generate SAI firing patterns in afferents [8]. To test that hypothesis *in silico*, we used our model to predict SAI firing with only SI and RI current components in the generator function (Fig 3, Table 1). We find that a model incorporating these two currents is able to replicate the SAI firing for the ramp and very early hold of the stimulus from 0–1 s, (Fig 3C, “No USI, 200 ms SI”). It also can replicate the slow adaptation of the sustained response to a certain degree. That said, in order to fit the slow adapting firing such that it does not plateau from about 1.5 to 5 sec, a larger SI current time constant is needed than the 200 ms recorded from isolated Merkel cells. Therefore, in order to correct the discrepancy, we tested two solutions: 1) extending the SI function’s time constant and 2) introducing a third USI generator current. In the former solution, extending the SI function’s time constant to 570 ms (Fig 3C, “No USI, 570 ms SI”) generated a predicted SAI firing pattern that reasonably well recapitulated the decay. However, as noted in the section below considering SAI firing in *Atoh1*^*CKO*^ mice, relying only on a RI time constant will be quite problematic. In the latter solution, introducing a third USI current, with a time constant of 1.7 s (Fig 3C, “USI, 200 ms SI”) likewise fit the recording data. The current that underlies the IFF responses of Fig 3C is shown in Fig 3G. Note that in another attempt to fit the time course of the decay in IFF response—without the USI current—the skin’s viscoelasticity was varied in a set of computational experiments, but could not achieve the time course of the decay (S4 Fig).

**Table 1:** Parameters of the generator function for the base case “Wildtype (with USI)” and two alternate cases, which illustrate the need for the USI current. Parameters for the *Atoh1*^*CKO*^ case are also shown.

	*τ*_*RI*_ (ms)	*τ*_*SI*_ (ms)	*τ*_*USI*_ (ms)	*K*_*SI_Peak*_	*K*_*SI_Steady*_	*a* (pA/Pa)	*b* (pA/Pa)	*c* (pA/Pa)
Wildtype (with USI)	8.0	200.0	1744.6	0.87	0.13	0.74	0.24	0.07
Wildtype (without USI, long SI	8.3	569.8	-	0.82	0.18	0.99	0.26	-
Wildtype (without USI)	8.0	200.0	-	0.81	0.19	0.74	0.36	-
*Atoh1*^*CKO*^	8.0	-	1744.6	-	-	0.74	-	0.07

### Sensitivity of parameters underlying slowly inactivating and rapidly inactivating currents

Several additional computational experiments were performed to vary combinations of parameters, though none impacted the issue of the plateau in the sustained phase. For example, small increases in the decay time constant of the RI current, *τ*_*RI*_, resulted in increased peak current amplitude during the ramp phase of stimulation (Fig 3D). Increasing the decay time constant of the SI current, *τ*_*SI*_, resulted in increased peak current amplitude during the early-hold phase (Fig 3E). Lastly, increasing the peak/steady-state ratio of the SI current, *K*_*SI_Peak*_, led to increased peak current amplitude during early-hold and late-hold phases (Fig 3F). Together, these experiments demonstrated that the model was sensitive to small changes in biological parameters, with a high degree of sensitivity. Note that each of the computational experiments in Fig 3D–3F (with corresponding IFFs in S2 Fig) was done with the USI current enabled.

### Predicting SAI firing in *Atoh1*^*CKO*^ mice

**Fig 4:**
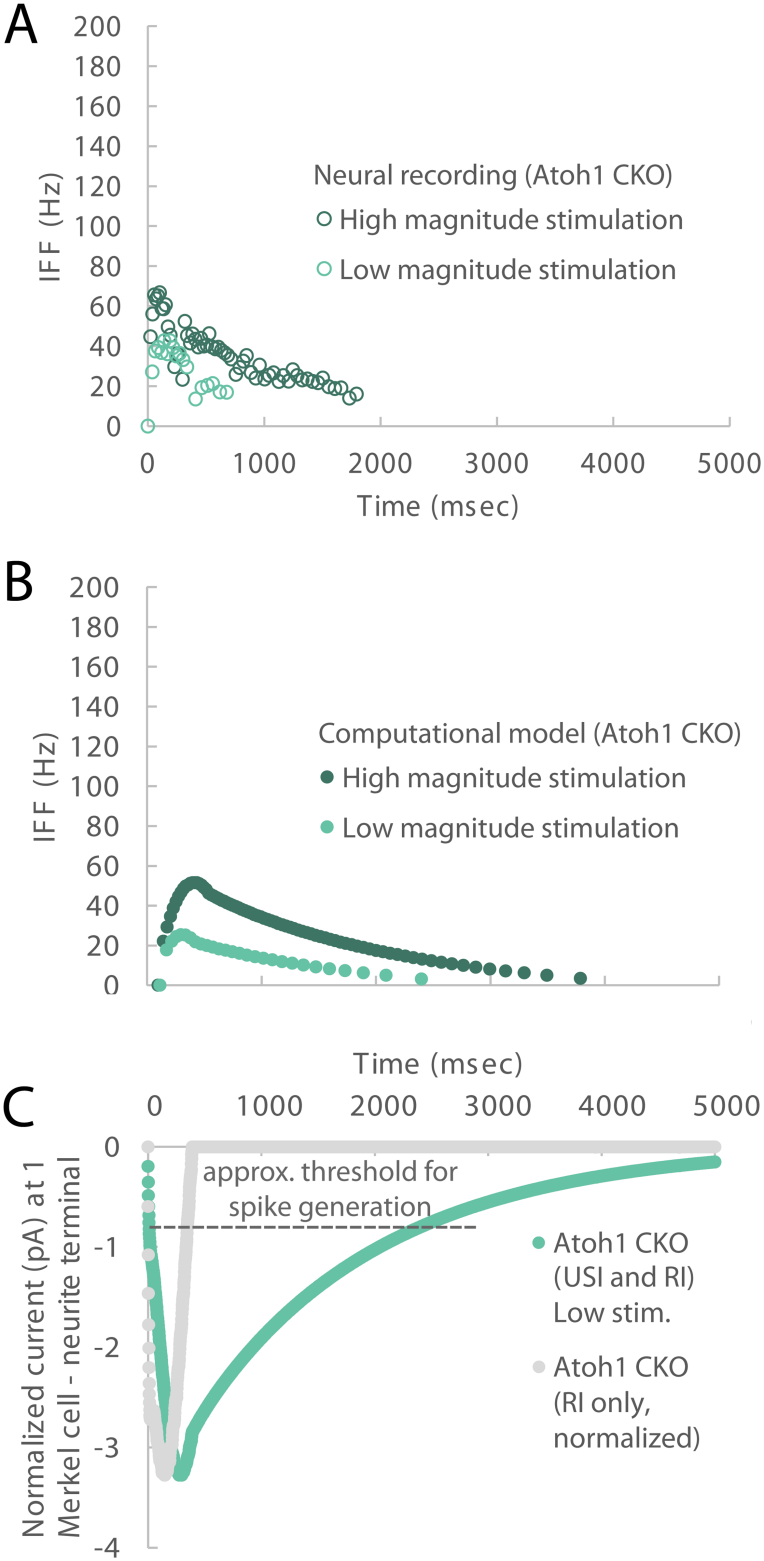
Modeled response for the case of Atoh1 knockout animals. By selectively deactivating sub-components of the generator function, IFF plots over time are generated (panel B) similar to those observed in electrophysiological recordings for Atoh1 knockout animals (panel A), in terms of attenuated temporal and spatial response compared to wildtype responses in Fig 3. Data from panel A were originally reported in Maksimovic, et. al. 2014 [3]. The two traces per plot represent two magnitudes of ramp and hold stimulation. Here, to model the Atoh1 knockout response, the SI component is removed entirely. In panel C, the current underlying the low magnitude stimulation case is shown in “Atoh1 CKO (USI and RI) Low stim.” As previously noted in panels C and G of Fig 3, an alternate approach to utilizing a USI term is to extend the duration of the SI term’s time constant. Aside from physiological feasibility, when the SI time constant is increased and USI not utilized, then the Atoh1CKO response—made up of USI and RI components—would revert to only an RI component (normalized to the magnitude of the USI and RI case) and its rate of decay does not match the Atoh1CKO current, panel C: “Atoh1 CKO (RI only, normalized).” In comparison to the electrophysiological recordings in panel A, its current decays to 0 IFF (where each line in panel C crosses the “approx. threshold for spike generation”) in 0.25 s whereas the IFF continues for 1–2 s in observed recordings. In fact, when the “Atoh1 CKO (RI only, normalized)” current is run through the full model, it produces just one spike.

Of the aforementioned model parameter solutions that afford slow adaptation in firing in the sustained phase for wildtype animals, that one which extends the time constant on the SI current is highly problematic for *Atoh1*^*CKO*^ animals for which there is only RI current. This situation substantially contributes to the argument for the inclusion of the USI term. In particular, Merkel cells are required for canonical SAI responses in mice [3,4]. Mice that lack either Merkel cells entirely (*Atoh1*^*CKO*^), or *Piezo2*, the principal mechanosensitive ion channel in Merkel cells (*Piezo2*^*CKO*^), exhibit truncated SAI firing in response to mechanical stimulation. In order to predict SAI firing in *Atoh1*^*CKO*^ mice, which lack Merkel cells but maintain touch-dome branching arbors, the model’s SI current was set to zero. With the USI current enabled, in the neurite along with the RI current, we are able to recapitulate the observed, truncated firing patterns in *Atoh1*^*CKO*^ mice (Fig 4). In particular, in alignment with the recorded data, the peak IFF in the simulated *Atoh1*^*CKO*^ mice was attenuated in magnitude, as compared to the wildtype animals. As well, the ramp and early decay of the IFF was attenuated in time, as compared to the wildtype animals, though the recording data does not elicit spikes at less than about 15 Hz. Note that the model’s free parameters were kept at the same values as when fitted to recording data from wildtype mice. Neither these nor the biologically derived parameters were modified in extending to the predictions for the *Atoh1*^*CKO*^ mice. The only change was in setting the SI current to zero, and noting the positioning of the USI current within the neurite due the anatomy of *Atoh1*^*CKO*^ mice.

**Fig 5:**
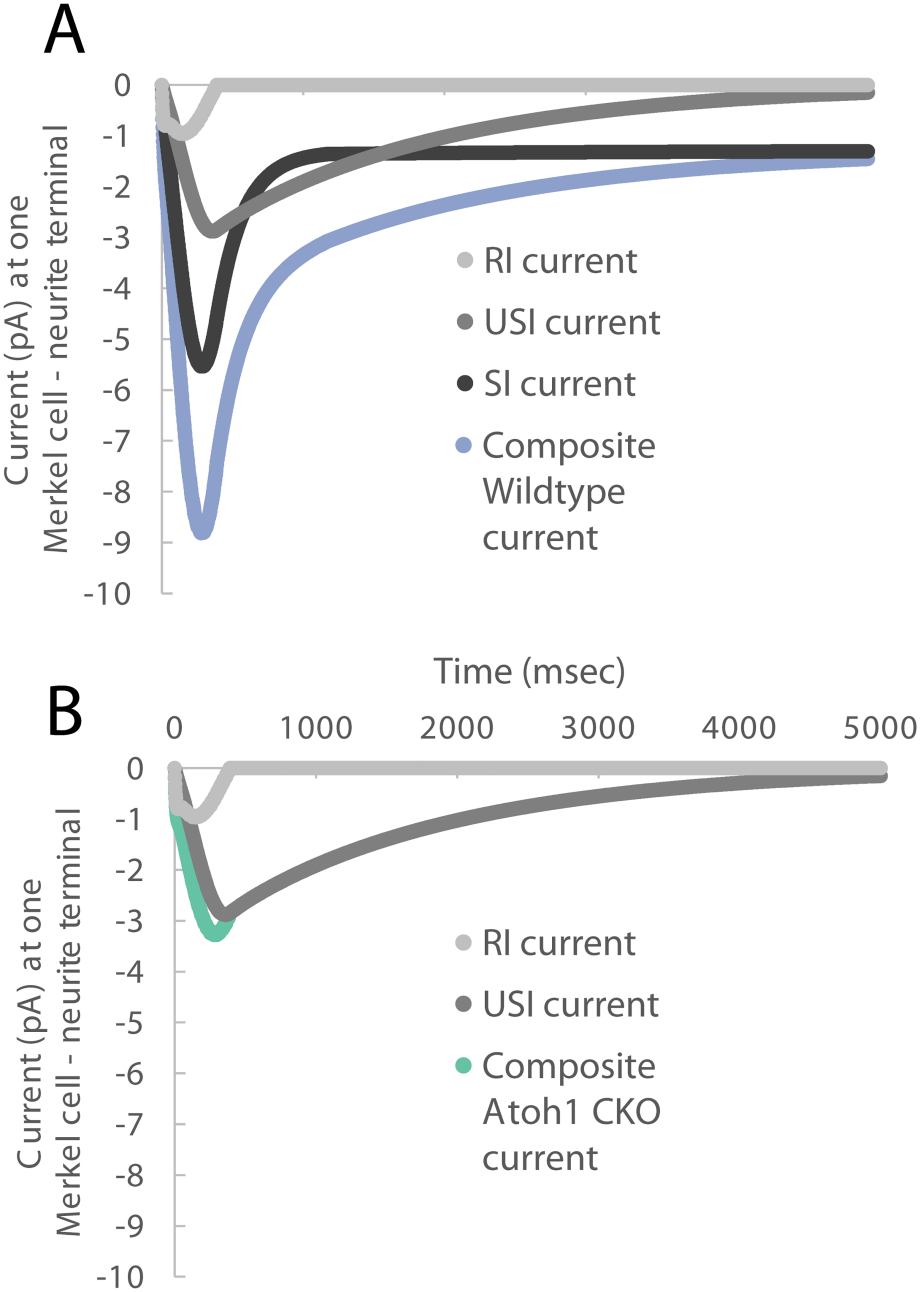
The respective currents underlying the composite current at one modeled Merkel cell—Neurite terminal, for wildtype (panel A) and *Atoh1*^*CKO*^ (panel B) mouse cases. Interestingly, across both animal types, the SI current is larger than the RI current, even in the dynamic phase of the stimulus. As well, the USI current is larger than the RI current. In the *Atoh1*^*CKO*^ case then, it is the USI current that is nearly entirely driving the response.

Finally, the relative relationships of the current values underlying the wildtype and *Atoh1*^*CKO*^ mice cases are shown in Fig 5. It is notable the magnitude of USI current is greater than RI current, in both wildtype and *Atoh1*^*CKO*^ mouse simulations. As well, the SI current is of larger magnitude than the RI current for the wildtype case, even during the ramp of the stimulus.

## Discussion

Computational modeling enables synthetization of diverse, biologically derived observations, in order to test hypotheses about complex processes. Here, we describe a computational model that is the first of its kind to employ physiologically derived parameters to further our understanding of the Merkel cell-neurite complex. Fundamental to this model is a generator function that converts interior stress in the skin to current at a single neurite. Importantly, the output of the model is a spike firing response that can recapitulate touch-evoked patterns observed to be characteristic of the SAI afferent, in particular, slow adaptation to the sustained hold of the stimulus. This capability is enabled by the computational combination of multiple generator currents, each on different timescales. However, it is only achievable by including an ultra-slowly inactivating current positioned in the sensory neuron. This addition is vital for differentiating the firing patterns observed between wildtype and *Atoh1*^*CKO*^ mice that lack Merkel cells and their associated ion channels.

### A generator function that is stimulus independent

Similar to the multi-level stratification in Johnson’s model [10], we included three input-output factors in modeling the SAI afferent: 1) surface stimuli propagates towards its interior layers (skin mechanics), 2) local tissue deformation is converted into current at neurites (generator function), and 3) generator current is converted into potential and the generation of spikes (neural dynamics). Previous attempts to model the Merkel cell-neurite complex have taken a simplifying approach, where the fundamental parameters of spike generation are directly tied and fitted to a presented stimulus. Additionally, these models do not rely on biologically derived parameters [9–15]. Such assumptions obscure the individual biophysical interactions of Merkel cells and neurites, and reduce the physiological relevance of these models. Specifically, the direct conversion of time derivatives of stimulus position into receptor current, used in these models, makes them non-physiologically based and heavily dependent upon parameter fitting to particular surface stimuli. For example, in a previous study that predicted the timing of individual spikes evoked by mechanical vibrations in three types of mechanoreceptive afferents [12], stimulus displacement and its derivatives (position, velocity, acceleration, and jerk) were separately filtered using different temporal linear filters and summed with different weights to form current input to a neural dynamics model. Similarly, in a focus on ramp-and-hold stimuli [5], stresses and strains within the skin were converted into receptor current. While perhaps a stress term represents a static response similar to the Merkel cell mechanism and its first derivative a dynamic response similar to the neurite mechanism, the mapping of such derivatives to either physiological mechanism is rudimentary and not clearly differentiated from what could also be framed as direct ties to stimulus position and movement.

In contrast, the model herein combines internal skin stress with fixed, experimentally observed time constants, to predict mechanically evoked Merkel-cell afferent firing. Furthermore, in transforming skin stress to current, the model incorporates a linear convolution over time, enabling a recent “biomechanical history” of the stimulus to be carried forward. While our model affords a means of storing the prior biomechanical history of the stimulus, via the present value of decaying current, there are alternative strategies to accomplishing this goal. For example, one could simply sum the three most recent timestamps of modeled current. However, such means of time-dependent storage is not a biologically relevant modeling strategy. Thus, the instantaneous linear convolution of skin stress more adequately reflects putative neurobiological mechanisms due to a carry-forward characteristic. This strategy provides a biologically relevant way of temporally generating and preserving generator current.

### The ultra-slowly adapting current

In order to recapitulate the experimentally observed touch-evoked firing patterns of Merkel-cell afferents, in particular the slow adaptation in firing in the sustained hold of the stimulus, our *in silico* analysis predicts that sensory neurons produce a USI generator current in addition to RI generator current. The USI current was essential in this model for replicating the truncated adaptation in firing for *Atoh1*^*CKO*^ animals, when the SI currents contributed by the Merkel cell are not present. Without the USI current, the existence of only RI current drives spike firing to zero too rapidly (about 0.25 s, Fig 4C) as compared to neural recording data (~1–2 s, Fig 4A). We have yet to observe a mechanosensitive USI current in Merkel-cell afferents, but this may be due to under sampling this rare neuronal population, or *in vitro* recording conditions. Although mechanically activated USI currents have primarily been observed in small-diameter, presumably nociceptive DRG somata, some groups have reported USI currents in putative LTMRs [20] [21]. Thus, we speculate that Merkel-cell afferents might express mechanosensitive USI generator currents. Alternatively, keratinocyte-derived signals might drive the USI current in the sensory neurons [22–24]. A third possibility is that voltage- or calcium-dependent currents that activate downstream of neuronal Piezo2 might account for the USI current. Although future experimental studies are needed to identify the origin of the USI current, a mechanism along these lines is playing a significant role, as Fig 5 denotes, the RI current plays a relatively smaller role.

Our findings raise the possibility that a complex interaction between Piezo2 ion channels and previously unsuspected conductances in sensory neurons govern firing at the Merkel cell-neurite complex. This work sets the stage to identify downstream molecular mechanisms, as well as enhances our understanding of the fundamental mechanosensory principles that govern tactile function and encoding in the nervous system.

In sensory systems, adaptation mechanisms work on multiple time scales to maintain sensitivity to dynamic stimuli. For example, light adaptation in vertebrate rod photoreceptors can be fit with a double exponential function whose fast time constant is in the range of the USI currents our models predict [25]. Thus, our models suggest that Merkel cell-neurite complexes, like other sensory receptors, employ multiple adaptive mechanisms that operate on different time scales. The relative contributions of these mechanisms to sensory signaling remain to be tested experimentally.

### Model assumptions and justification of fitting procedures

Our model depends on several underlying assumptions. First, we assumed that the overall generator current is simply the superposition of three individual generator current sources (SI, RI and USI). Second, as direct recordings from Merkel-cell neurites have not been reported in any species, we made a simplifying assumption that the current level and decay of the Merkel-cell dependent SI current in neurites is linearly related to the membrane potential recorded for Merkel cells under current-clamped conditions. A linear relationship was chosen because more complex transformations are not warranted based on the available biological data. Third, we assumed that the current level and decay rate of the neurite based RI current are similar to those recorded from rapidly inactivated DRG neurons *in vitro*, which correspond to low-threshold mechanoreceptors. Fourth, we have only modeled a single Merkel cell to single neurite interaction. However, Merkel cell-neurite complexes can connect in both chains and clusters [5,26], and during skin renewal Merkel cells and neurites undergo dynamic architectural remodeling [27]. It will be critical to integrate these additional complexities in future modeling studies to better understand how tactile information is coded.

### Skin viscoelasticity

A final note regards the relationship between the skin’s time-dependent viscoelasticity and the ultra-slowly inactivating current. Our computational effort includes a finite element model to account for the skin’s mechanics, which exhibit non-linear behaviors of hyperelasticity and viscoelasticity. The material properties in the finite element model were directly obtained from skin measurements made across a range of animals spanning stages of the mouse hair cycle [17,18]. The model utilizes clamped displacement stimuli, identical to those of the electrophysiological experiments [3]. Upon indentation into the skin, force at and stress around the stimulus tip is observed. As our prior validation indicates, model output well matches observations of tip force as it relaxes over time [16].

One might wonder if the issue of slow adaptation to a sustained stimulus (Fig 3C) might be accounted for by greater viscoelastic relaxation of the skin. To address this topic, simulations where the relaxation is varied over a biologically observed range are presented in S4 Fig. The results indicated that the time course of the decay in the spike firing could not be achieved by varying skin viscoelasticity alone. Even in the most extreme case, the model begins to yield an intermediately adapting response. Furthermore, even if the stress trace was inaccurate, considerably more change in stress (over a duration of about 1–2 s) would be required to influence RI current (given its 8 ms time constant) in order to recapitulate the *Atoh1*^*CKO*^ response (Fig 4). As well, given the constitution of the generator function, when stress decreases, current decreases. In fact, a paradoxical slow increase in stress following the stimulus ramp would be required to make-up for the absence of USI current.

Skin mechanics are simulated here by bulk tissue layers and do not include the micro-level mechanics of the touch dome. The micromechanics of touch domes have not been investigated, to our knowledge; however, several anatomical features suggest that the touch domes mechanical properties might differ from other skin areas. For example, touch domes contain a highly vascularized dermis, a thickened epidermis and a thin stratum corneum compared with adjacent skin regions [28]. Moreover, touch domes are marked by columnar keratinocytes and Merkel cells, whose intermediate filament cytoskeletons are molecularly distinct from that of surrounding epidermal keratinocytes [29,30]. Little is known about how specific cytokeratin isoforms contribute to skin mechanics [31]; however, a recent study has shown that, along the human hair follicle, mechanical stiffness changes with the organization of keratin networks [32]. Thus, future work is needed to determine whether touch domes have specialized tissue mechanics and, if so, how they might contribute to neuronal firing patterns.

## Materials and methods

### Mathematical form of the generator function

The generator function is a convolution of compressive stress interior to the simulated skin and three exponential functions that describe how a single Merkel cell-neurite complex responds to a step stimulation input with an instantaneous increase or decrease proportional to stress magnitude followed by an exponential decay. Electrophysiological recordings suggest that current in a neuron rapidly decays with a step stimulation, and we assume that a neurite behaves similarly. Therefore, the rapidly inactivating (RI) current in Eq 1 corresponds to decay time constant *τ*_*RI*_ and linear transformation coefficient *a*. The slowly inactivating (SI) current in Eq 2 corresponds to decay time constant *τ*_*SI*_, linear transformation coefficient *b*, and two ratio parameters *K*_*SI_Peak*_ and *K*_*SI_Steady*_, representing the peak and steady portions of a decaying trace (where *K*_*SI_Peak*_ + *K*_*SI_Steady*_ = 1). The ultra-slowly inactivating (USI) current in Eq 3 corresponds to decay time constant *τ*_*USI*_ and linear transformation coefficient *c*. Note that linear transformation coefficients *a*, *b*, *and c* serve to convert instantaneous stress to instantaneous current, linearly.

$$a\;*\text{exp}\;\left(-\frac{t-x}{\tau_{RI}}\right)$$ (1)

$$b\;*\;(K_{SI\_Peak}\;*\text{exp}\;\left(-\frac{t-x}{\tau_{SI}}\right)+K_{SI\_Steady})$$ (2)

$$c\;*\text{exp}\;\left(-\frac{t-x}{\tau_{USI}}\right)$$ (3)

Bringing Eqs 1–3 into the bracket of Eq 4, the complete form of the generator function is a convolution of these terms and the first derivative of stress input *σ* over time:

I(t)=∫x=0t[a*exp(-t-xτRI)+b*(KSI_Peak*exp(-t-xτSI)+KSI_Steady)+c*exp(-t-xτUSI)]*dσdxdx (4)

where *I* is the output generator current, *t* is time, and *x* is a variable of the integral. We use 0 as the mathematical baseline of *I*, and set it to 0 when it becomes negative. The terms *a* and *b* are instantaneous values while $\frac{d\sigma }{dx}$ along with the integral represents their decay over time, which is the means of storing the prior history of the stimulus, via the present value of receptor current, in a decaying fashion.

### Generator function in context of SAI whole end organ model

Since we cannot directly measure the receptor currents in a neurite that would emerge from the contribution of Merkel cell and neurite mechanisms, the generator function was validated in the context of an end-organ model for the SAI afferent [5]. In this model, one Merkel cell and its connecting neurite form a Merkel cell-neurite complex, where multiple complexes are clustered per heminode. For example, the end-organ structure includes 4 heminodes and therefore 4 clusters, with 3, 1, 5, and 8 Merkel cell-neurite complexes in each, noted as a {8, 5, 3, 1} structure (sequence does not matter). In each Merkel cell-neurite complex, a finite element model of the skin’s layers outputs compressive stress in to the skin given a stimulus input of displacement with a linear decelerating ramp-up. Different from prior work [11], a refined finite element model was used that was both hyper and viscoelastic as based directly upon measurements of the mouse, and using the output of maximum compressive stress instead of strain energy density components [16]. Each layer of the multi-layered model was represented by axisymmetric hybrid elements of quasi-linear viscoelastic material with Ogden and Neo-Hookean hyperelastic. In response to indentation by ramp-and-hold stimuli, its output of compressive stress over time is passed to the generator function, which calculates receptor current for one Merkel cell-neurite complex. Then, receptor current is multiplied by the number of Merkel cell-neurite complexes in a cluster as the total current entering the heminode, which is taken in a leaky integrate-and-fire model to accumulate enough potential to elicit a spike. There is therefore one LIF model at each heminode. Once the potential at a heminode reaches the firing threshold and elicits a spike, the potentials at other heminodes are immediately reset to baseline, and a refractory period of 1 ms is set. The parameters *R*, *C*, and *V* (resistance, capacitance, and firing voltage threshold) of the LIF model are set to 5 GΩ, 30 pF, and 30 mV, and are the same for all 4 LIF models in the model.

### Deriving and fitting the parameters of the generator function

The generator function utilizes eight parameters. Five biologically-derived parameters (*τ*_*RI*_, *τ*_*SI*_, *τ*_*USI*_, *K*_*SI_Peak*_ and *K*_*SI_Steady*_) were obtained from experimental data. Three free parameters (*a*, *b*, *and c*) were model fitted in the context of simulating an entire end organ.

Regarding the biologically derived parameters, time constant *τ*_*RI*_ in the neurite mechanism was fitted to the decay time constant obtained from the current recorded in the neuron of a whole cell over time under a voltage clamped prep, with step mechanical stimulation [19]. We assume similarity of current decay between such neurons and simulated neurites herein. A characteristic recording and its fitted trace are shown in S1B Fig. As shown in Table 2, a total of 44 measurements from nine preps were fitted using a single exponential decay functions of the form $y\;=\;a\;*\;\text{exp}\;(-\frac{x}{\tau })$. The mean value of all fitted time constants, 0.008 s, was used for *τ*_*RI*_, and the mean value ± standard deviation of all fitted time constants, 0.001 and 0.015 s, were used in numerical experiments with parameter changes. In contrast, the time constant *τ*_*SI*_, as well as the peak to steady state ratio parameters *K*_*SI_Peak*_ and *K*_*SI_Steady*_ of the Merkel cell mechanism were generated directly from single isolated Merkel cells. In this case, however, membrane potential over time was recorded in the current clamped prep [3]. We assume that the Merkel cell’s transmission mechanism most likely behaves like a synapse where changes in the cell membrane’s potential are linearly related to post-synaptic current under a step stimulation. A characteristic recording and its fitted trace shown in S1A Fig. A total of 12 voltage measurements from three Merkel cells were fitted using a similar single exponential decay plus a constant function, of the form $y\;=\;a\;*\text{exp}\;\left(-\frac{x}{\tau }\right)+b$. The mean value of all fitted time constants (Table 3), 0.2 s, was used for *τ*_*SI*_, and the mean value ± standard deviation of all fitted time constants, 0.05 and 0.35 s, were used in numerical experiments with parameter changes. *K*_*SI_Peak*_ and *K*_*SI_Steady*_ (Table 4) are dependent of each other, and have a sum of 1. Their values were obtained through numerical optimization, and falls within the range of the data from Table 1. Furthermore, the *τ*_*USI*_ parameter was tied to the time constant of 1.7 ms, found for putative LTMRs [20] [21], though it was slightly model fit around that value, using the procedure as noted below.

**Table 2:** Time constants in milliseconds for fitted traces of current recordings in the SAI afferent in response to a step mechanical stimulation near the end organ (fitting *τ*_*RI*_: mean = 8 ms, stdev = 5 ms).

	Run 1	Run 2	Run 3	Run 4	Run 5	Run 6	Run 7	Run 8	Run 9
Fiber 1	8.2								
Fiber 2	5.4	5.6	10.4	11.4	8.2	8.9			
Fiber 3	31.3	15.8	14.8						
Fiber 4	9.6	11.0	17.1						
Fiber 5	7.5	4.3	2.7	2.8	3.1	3.9	3.2		
Fiber 6	4.8	4.5	7.3	5.4	9.5	5.8			
Fiber 7	4.7	2.6	5.2	2.7	3.7	6.4	4.0	5.6	4.3
Fiber 8	3.4								
Fiber 9	5.9	4.4	6.9	15.0	9.9	6.1	7.9	14.5	

**Table 3:** Time constants in milliseconds for fitted traces of potential recordings for isolated Merkel cells in response to a step mechanical stimulation (fitting *τ*_*SI*_: mean = 200 ms, stdev = 150 ms).

	Run 1	Run 2	Run 3	Run 4	Run 5
Fiber 1	163.7	555.6	288.2	387.6	
Fiber 2	42.8	50.3	342.5	30.0	43.0
Fiber 3	7.2	4.0	7.9		

**Table 4:** Ratio of potential in Merkel cell recordings from the peak value to the steady state value in response to a step mechanical stimulation (fitting *K*_*SI_Peak*_).

	Run 1	Run 2	Run 3	Run 4	Run 5
Fiber 1	0.41	1.00	0.88	1.00	
Fiber 2	0.19	0.33	1.00	0.36	0.50
Fiber 3	0.21	0.26	0.27		

Free parameters *a*, *b*, and *c* represent the linear transformation from instantaneous stress to instantaneous current for RI, SI, and USI components, respectively, and their true values are not presently measurable. In particular, the magnitude of one mechanism relative to another, as well as the way in which the Merkel cell current is transferred to the neurite and mixes with it are unknown. Therefore, they are fitted in the context of simulating an entire end organ. Ultimately they are set at 0.74, 0.24, and 0.07, respectively, such that the RI current in the neurite is more sensitive than the SI current in the Merkel cell, and both are far more sensitive than the USI current.

Before delving into further details of parameter and model fitting, we note several relationships among the parameters of the generator function. First, increasing the magnitudes of parameters *τ*_*RI*,_ *τ*_*SI*_ *and K*_*SI_Peak*_ (decaying time constants and peak/steady ratio) independently increase receptor currents in ramp-up, early-hold, and late-hold phases, respectively, as noted (Fig 3D, 3E and 3F). The ratio of these parameters can also change the ratio of the firing rates in different phases of the stimulus. For example, decreasing *τ*_*RI*_ will decrease the overall ramp-up firing rate magnitudes only, and therefore can result a decrease of the ramp-up:late-hold firing rate ratio. As well, decreasing *a*:*b* from 3.08 to, say, 1:1, will decrease the firing rate ratio of ramp-up:late-hold. Finally, the ratio of *a*:*b*, though at present not constrainable by biopotential measurement, could potentially be constrained in the future by either spike or current recordings at the neuron by comparing Piezo2 deficient and wildtype mice.

The model fitting procedure and its justification is as follows. As typical values of currents in whole afferent recordings can reach up to 250 pA, and that our model contains 17 Merkel cell-neurite complexes to achieve this value, we estimated the receptor currents from a single Merkel cell-neurite complex should be evenly divided by 17, with a peak value of about 10–20 pA. With this as a starting point, their values were fitted in the whole end-organ model so that firing rates predicted over the ramp-up, early-hold, and late-hold phases of the stimulus mimic the electrophysiological recordings in Table 2, for an afferent described elsewhere [16] with the indenter tip size adjusted from 1 to 1.5 mm in diameter. Specifically, the experimentally recorded spike timings were first converted to instantaneous firing rates, and then smoothed with a moving average with window width of 5. Then, all smoothed firing rates were logarithmically sampled (a total of 50 data points) to put higher weight of the early-hold phase during the fitting. Finally, the Levenberg-Marquardt method was used to numerically maximize the coefficient of determination, calculated by interpolating the modeled firing for comparison to the smoothed firing from recordings [33]. Two stimulus displacements were fitted and we averaged the parameters from these two fits to obtain the final values, which are 0.74, 0.24, and 0.07 pA/Pa, for a, b, and c respectively.

## Supporting information

S1 Fig The parameters *τ*_*SI*_ and *τ*_*RI*_, respectively, of the generator function are fitted from recording data. In particular, panel (A) shows a characteristic trace of *in vitro* Merkel cell membrane potential over time under a current clamped prep, delivered a step mechanical stimulus at about 88% of the saturation threshold, and panel (B) shows a characteristic trace of current recorded in the DRG neuron of a whole cell over time under a voltage clamped prep, delivered a mechanical stimulus of about 85% of the saturation threshold. The movement and hold of the stimulus are shown to the top of each figure by the dark and light areas, respectively.
(TIF)

S2 Fig Model produced instantaneous firing frequencies under a parameter sweep with computational model, low magnitude of stimulation, wildtype case. Panels A—C show IFFs when the generator function is run in the context of the entire end organ model. These correspond to the parameter modifications to generate the currents in Fig 3, panels D—F. The tau values are in units of ms. See the Fig 3 caption regarding parameters and impact.
(TIF)

S3 Fig To accompany the low magnitude stimulus example in Fig 3C, shown here is the high magnitude stimulus case, likewise showing the need for the USI component. Without the USI component, the output IFF reaches a plateau and does not adapt as is typically observed for SAI afferents.
(TIF)

S4 Fig Time course of IFF decay observed in neural recordings cannot be achieved by skin viscoelasticity alone in absence of USI current. In Panel A, three computational simulations were run where the skin’s viscoelasticity was varied by changing G_∞_ from 0.81, 0.35, and 0.10 for a 418 micron thick skin in the finite element model. The range of relaxation simulated follows from taking the maximum, median, and minimum values of prior measurements done over a large cohort of animals [17]. Note this work had shown the time constants of skin relaxation to be positively correlated with the steady-state residual stress ratio (G_∞_) and have the same effect in reducing the time constant. The time constants were therefore fixed at the same order of magnitude, namely the median value from the aforementioned prior work, in particular τ1 = 0.08 s, τ2 = 1.21 s. The three stress traces from Panel A were input to the whole end organ neural model, with the USI current term disabled, and the resultant IFF decay traces are shown in Panel B, in the context of the corresponding neural recording. As is observable, the time course of the decay in the spike firing could not be achieved by varying skin viscoelasticity alone. Neural reocrdings in panel B were originally reported in Maksimovic, et. al. 2014 [3].
(TIF)
