# Supplementary figures and images for: Computation predicts rapidly adapting mechanotransduction currents cannot account for tactile encoding in Merkel cell-neurite complexes
Source: PLoS Comput Biol. 2018 Jun 29;14(6):e1006264. doi: 10.1371/journal.pcbi.1006264 (PMC6042796; doi:10.1371/journal.pcbi.1006264)

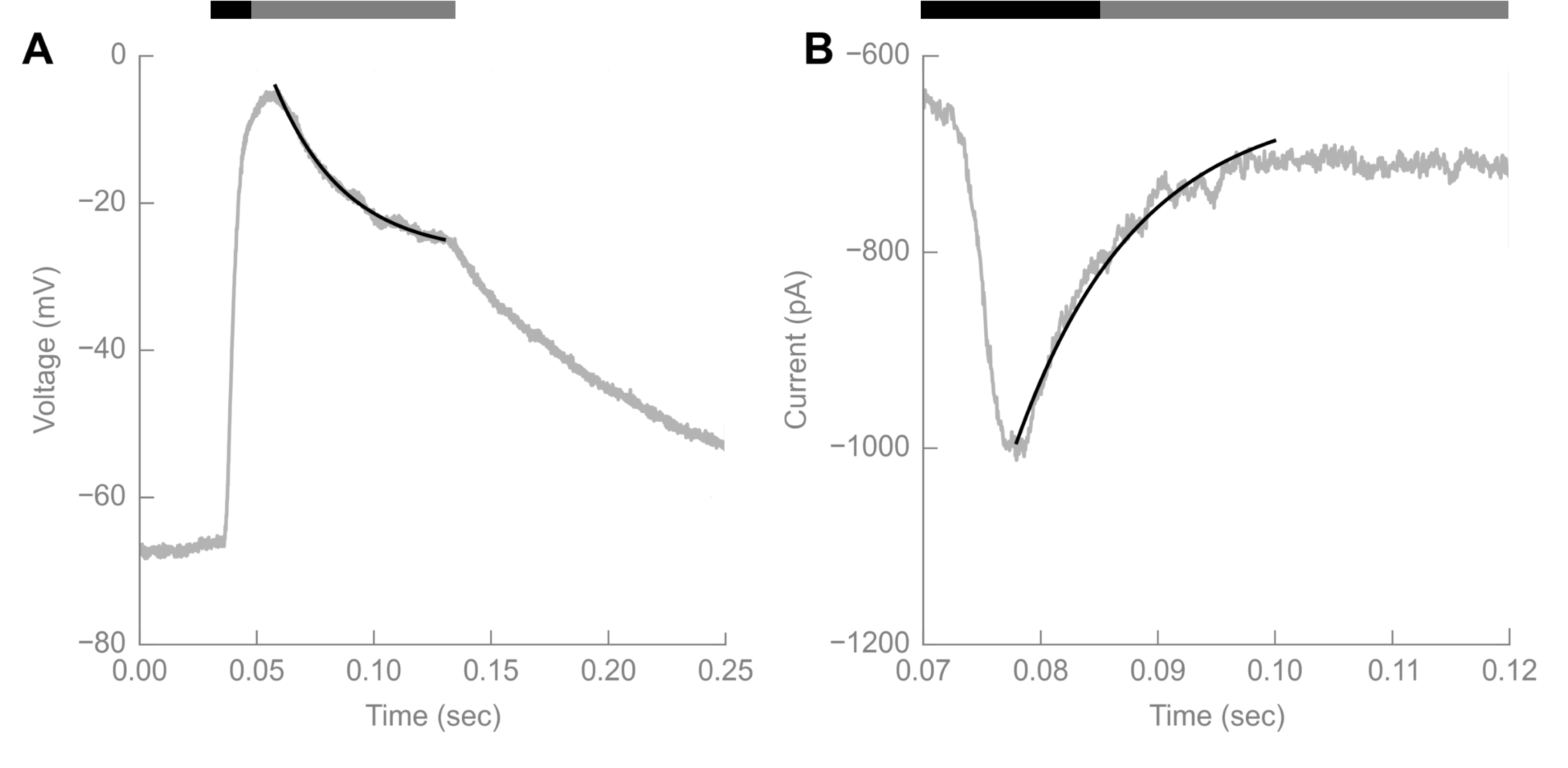

Supplement: S1 Fig — In particular, panel (A) shows a characteristic trace of in vitro Merkel cell membrane potential over time under a current clamped prep, delivered a step mechanical stimulus at about 88% of the saturation threshold, and panel (B) shows a characteristic trace of current recorded in the DRG neuron of a whole cell over time under a voltage clamped prep, delivered a mechanical stimulus of about 85% of the saturation threshold. The movement and hold of the stimulus are shown to the top of each figure by the dark and light areas, respectively. (TIF) [file pcbi.1006264.s001.tif]

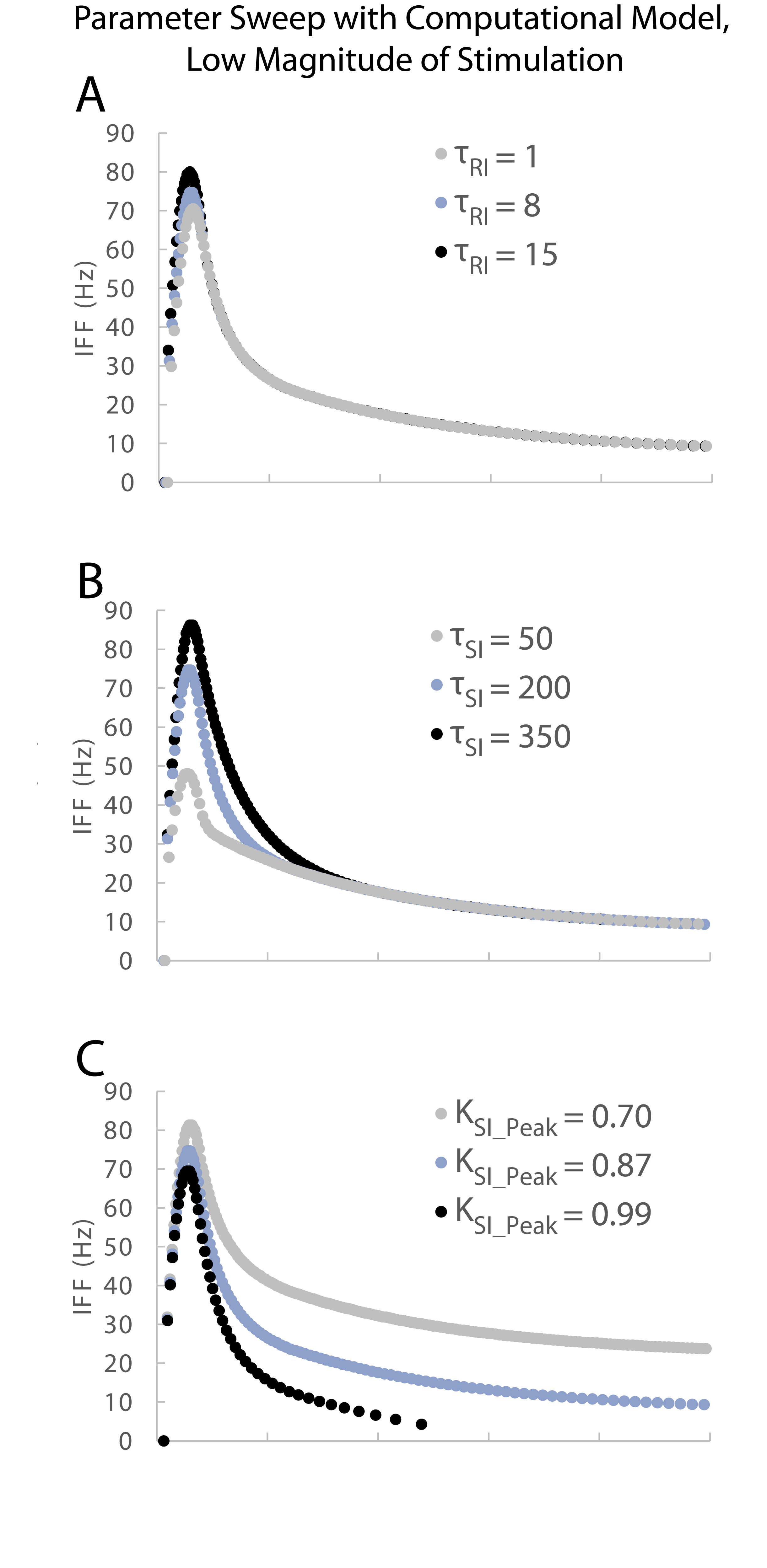

Supplement: S2 Fig — Panels A—C show IFFs when the generator function is run in the context of the entire end organ model. These correspond to the parameter modifications to generate the currents in Fig 3, panels D—F. The tau values are in units of ms. See the Fig 3 caption regarding parameters and impact. (TIF) [file pcbi.1006264.s002.tif]

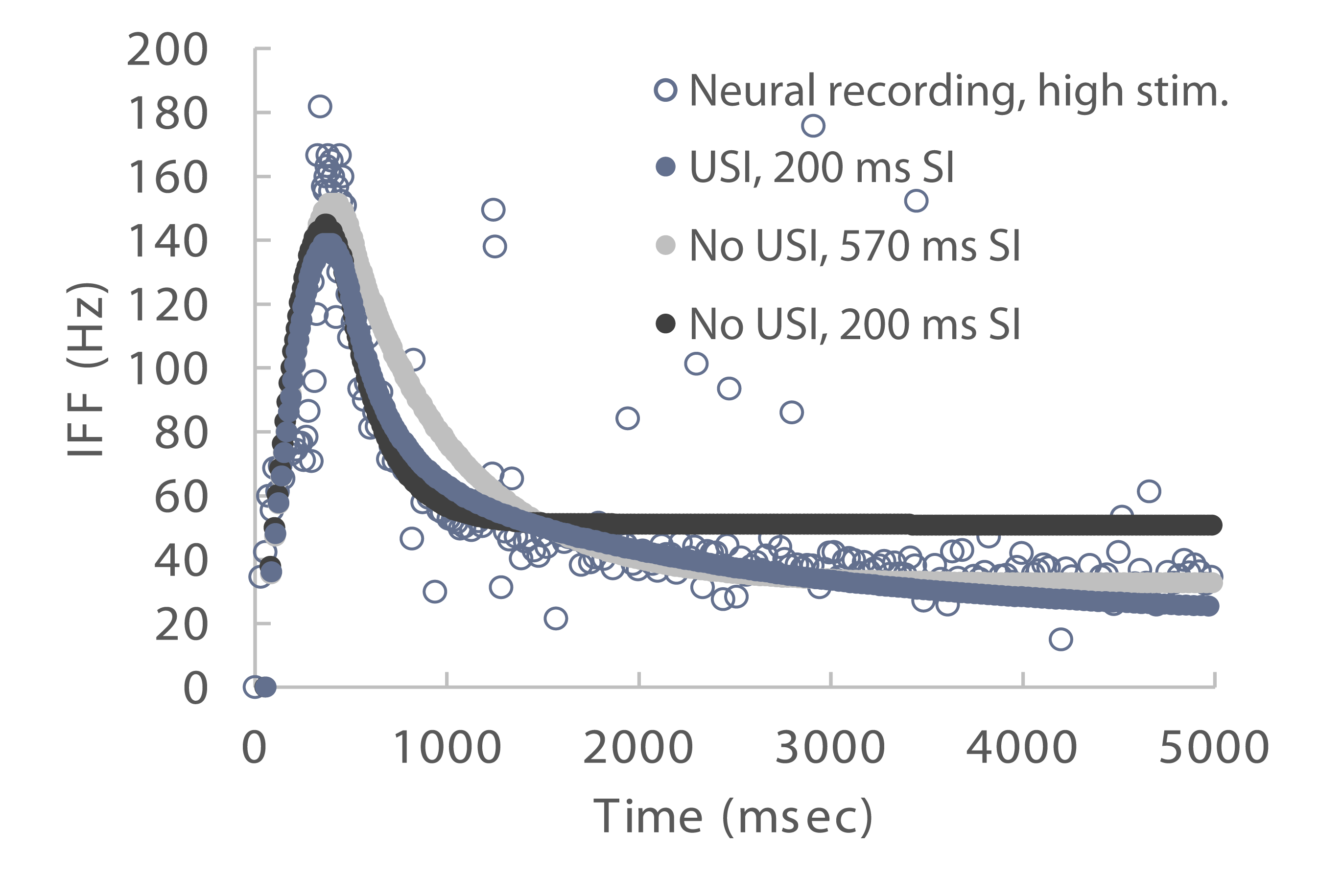

Supplement: S3 Fig — Without the USI component, the output IFF reaches a plateau and does not adapt as is typically observed for SAI afferents. (TIF) [file pcbi.1006264.s003.tif]

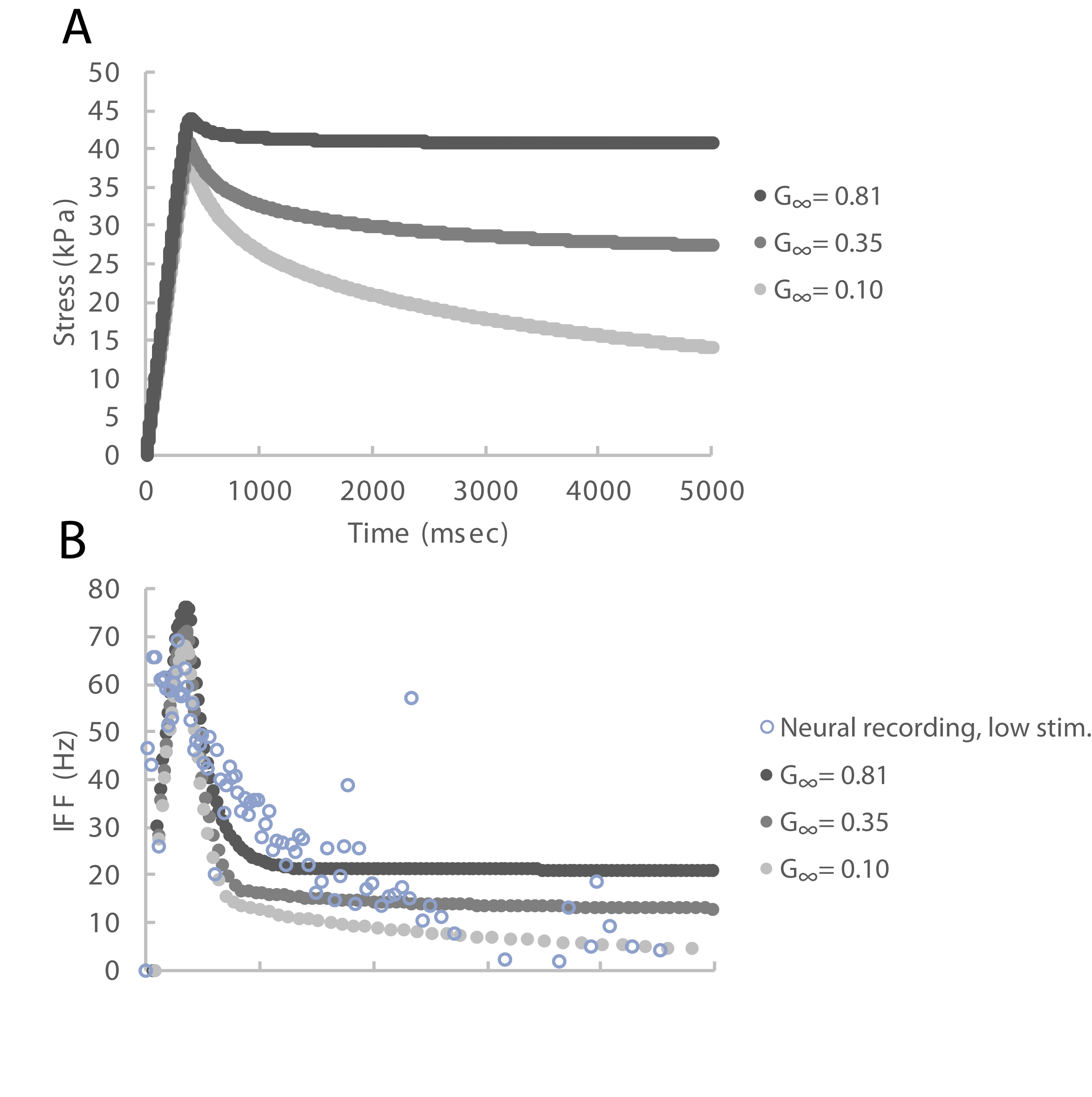

Supplement: S4 Fig — In Panel A, three computational simulations were run where the skin’s viscoelasticity was varied by changing G∞ from 0.81, 0.35, and 0.10 for a 418 micron thick skin in the finite element model. The range of relaxation simulated follows from taking the maximum, median, and minimum values of prior measurements done over a large cohort of animals [17]. Note this work had shown the time constants of skin relaxation to be positively correlated with the steady-state residual stress ratio (G∞) and have the same effect in reducing the time constant. The time constants were therefore fixed at the same order of magnitude, namely the median value from the aforementioned prior work, in particular τ1 = 0.08 s, τ2 = 1.21 s. The three stress traces from Panel A were input to the whole end organ neural model, with the USI current term disabled, and the resultant IFF decay traces are shown in Panel B, in the context of the corresponding neural recording. As is observable, the time course of the decay in the spike firing could not be achieved by varying skin viscoelasticity alone. Neural reocrdings in panel B were originally reported in Maksimovic, et. al. 2014 [3]. (TIF) [file pcbi.1006264.s004.tif]
